# The impact of core self-evaluations and person-job fit on work-related outcomes

**DOI:** 10.3389/fpsyg.2024.1341717

**Published:** 2024-02-21

**Authors:** Zongguo Zhang, Zhen Yan

**Affiliations:** ^1^Qingdao Vocational and Technical College of Hotel Management, Qingdao, China; ^2^North-Chiang Mai University, Chiang Mai, Thailand

**Keywords:** Core self-evaluations, person-job fit, emotional exhaustion, organizational dehumanization, service recovery performance

## Abstract

**Introduction:**

The aim of this study is to explore the mediating effect of emotional exhaustion (EE) between core self-evaluations (CSE), person-job fit (PJ fit) and service recovery performance (SRP). The current research also examines whether organizational dehumanization (OD) moderates the relationship between two antecedents, EE and SRP.

**Methods:**

We collected time-lagged and multi-source data from star-rated hotels in three cities in China. In particular, direct supervisors of frontline employees (FLEs) rated their SRP (*N* = 32 supervisors and their 212 immediate employees). The hypotheses were gauged via PROCESS (version 3.4).

**Results:**

The results indicate that CSE and PJ fit alleviate FLEs’ EE, which subsequently advances their SRP. OD moderates the direct impact of two antecedents on EE and SRP. Moreover, OD moderates the indirect influence of two antecedents on SRP through EE.

**Discussion:**

The hospitality literature currently lacks an in-depth understanding of the underlying mechanism through which CSE and PJ fit affect FLEs’ SRP. This study incorporates EE as a mediator of the CSE’s and PJ fit’s impact on SRP, and to the authors’ knowledge, this is the first attempt to evaluate whether OD moderates the direct influence of CSE and PJ fit on SRP and the indirect impact of CSE and PJ fit on the aforementioned outcome via EE.

## 1 Introduction

Hospitality organizations aspire to achieve flawless service delivery, yet attaining such perfection proves exceedingly challenging. Even renowned firms acknowledged for delivering exceptional service may experience service failures (Kim and Baker, 2020). Service failure, a scenario in which service performance does not meet customer expectations, is a pervasive occurrence in the in the process of providing service (Zahoor, 2020). The actions taken by a service provider in answer to such a service failure are termed “service recovery” in the service literature. It’s worth emphasizing that effectively executing service recovery can yield a range of advantages. These encompass strengthened positive word-of-mouth promotion and heightened customer loyalty, resulting in increased economic benefits for hospitality organizations (Choi C. H. et al., 2014; Choi Y. et al., 2014; Yunus, 2023).

Existing studies provide valuable insights into job-related antecedents of service recovery performance (SRP), including factors like job demands (e.g., customer-related social stressors, workplace ostracism) (Choi C. H. et al., 2014; Choi Y. et al., 2014; Paşamehmetoğlu et al., 2022) and job resources (e.g., green human resource management, co-worker and supervisor support) (Guchait et al., 2014; Iftikhar et al., 2021). It is crucial to acknowledge that frontline employees’ (FLEs) SRP is influenced not only by job-specific demands and resources, but also by their own personal resources (Van Vaerenbergh and Orsingher, 2016). Based on conservation of resources (COR) theory, core self-evaluations (CSE) is recognized as personal resources and person-job fit (PJ fit) as conditional resources (Lee, 2015; Yan et al., 2022). These pivotal resources empower employees to adeptly navigate challenging circumstances, effectively cope with adversity and stress, ultimately culminating in workplace success (Kim et al., 2017). As such, CSE and PJ fit are posited to effectively enhance employees’ SRP in the hospitality industry.

Emotional exhaustion (EE) refers to the state of feeling emotionally overextended and exhausted due to one’s work, and it has been demonstrated to have a detrimental impact on job attitudes and behaviors (Maslach and Jackson, 1981). The pervasive effect of EE is particularly evident in the hospitality industry, where FLEs engage in numerous customer interactions which can significantly influence their attitudes and performance (Choi C. H. et al., 2014). According to COR theory, when FLEs experience EE, they may struggle to maintain satisfactory SRP due to a depletion of essential resources. Consequently, FLEs may respond negatively by reducing their SRP in order to prevent further resource depletion. Prior studies have also shown that personal and conditional resources can be effective in alleviating EE especially in the hospitality field (Chen and Eyoun, 2021; Yan et al., 2023). Hence, we employ EE to explore the underlying mechanism linking CSE, PJ fit, and SRP.

In modern hospitality sector, where work tends to be standardized, service employees are often treated as mere commodities traded in the workplace (Gip et al., 2023). When organizations manage FLEs through rigid procedures, treating them as if they were machines and disregarding their basic human needs, employees may perceive this as an act of organizational dehumanization (OD) (Gip et al., 2023). OD has become a prevalent stressor for hospitality FLEs (Muhammad and Sarwar, 2021). From the viewpoint of COR theory, CSE and PJ fit as personal and conditional resources can effectively alleviate FLEs’ stressor and strain such as OD and EE in this study. Furthermore, previous research on OD has examined its attitudinal and behavioral outcomes, such as organizational commitment, job satisfaction, EE, turnover intentions and performance in the context of hospitality (Sarwar and Muhammad, 2020; Muhammad and Sarwar, 2021; Gip et al., 2023). Thus, OD may play a moderating effect between CSE, PJ fit, EE and SRP.

The current empirical study delves into the aforementioned relationships and addresses three important research gaps. First, it explores how personal and conditional resources serve as effective remedies in mitigating EE. Specifically, CSE and PJ fit as personal and conditional resources have consistently emerged in various studies (Lee, 2015; Dong et al., 2020; Raza et al., 2023), demonstrating their capacity to enhance service quality orientations (Yavas et al., 2010). However, to date, no empirical research has examined the direct relationships of CSE and PJ fit with SRP. Second, while an abundance of empirical studies in the extant literature have proved that CSE and PJ fit can relieve FLEs’ emotional exhaustion from the perspective of COR theory (Mulki et al., 2006; Anand and Mishra, 2021), similar studies within the hospitality industry remain scarce. Finally, OD has been frequently used as an antecedent in many hospitality studies (Muhammad and Sarwar, 2021; Gip et al., 2023), nevertheless, it has never been utilized as a moderating variable between CSE, PJ fit and performance outcome. What’s more, OD has shown significant associations with various resources (Nguyen and Stinglhamber, 2021; Scrimin and Rubaltelli, 2021) and performance (Gip et al., 2023). Thus, OD has been identified as key moderating variable in the current research, which deserves more attention from both hospitality researchers and practitioners.

To summarize, the research questions of the current study are posed based on the discussion above:

- Do CSE and PJ fit have effect on SRP?
- Are there mediating effects of EE on the relationships between CSE, PJ fit and SRP?
- Are there moderating effects of OD on the relationships between CSE, PJ fit, EE and SRP?
- Are there indirect effects of CSE and PJ fit on SRP via EE which are conditional on OD (Testing the moderated mediation effects)?

Grounded in this backdrop, this study aimed to address these questions by examining the effects of CSE and PJ fit on SRP, the mediating effect of EE and the moderating effect of OD among FLEs in hotel sector in China.

## 2 Literature review and research hypotheses

### 2.1 Core self-evaluations and service recovery performance

Service recovery delineates the strategic interventions undertaken by a service-oriented entity in response to instances of service failure, wherein a customer undergoes a substandard or inequitable service encounter. The primary objective is the restoration of customer contentment, thereby securing their allegiance to the establishment (Choi C. H. et al., 2014). As previously elucidated, the adept execution of service recovery initiatives bequeaths manifold advantages, encompassing the augmentation of favorable word-of-mouth endorsements and heightened patronage, thus conferring distinct competitive advantages upon the enterprise (Casidy and Shin, 2015; Akinci and Aksoy, 2019). Within the domain of the hospitality industry, SRP assumes an imperative role in job-related proficiency. It connotes FLEs self-perceptions of their own competencies and actions in rectifying instances of service shortcoming, ultimately culminating in customer satisfaction (Yadav and Dhar, 2021).

Judge et al. (1997) introduced the concept of CSE to elucidate the psychological mechanisms that underlie dispositional influences on employee outcomes, including job attitudes and job performance. Judge and Bono (2001) specifically defined CSE as basic conclusions or bottom-line evaluations about themselves. Thus, CSE represents the fundamental assessments that individuals form regarding their own capacities, worth, and competency. Furthermore, CSE encompasses four distinct yet interconnected personal traits: self-esteem emotional stability, locus of control, and generalized self-efficacy (Judge et al., 2005).

The fundamental tenet of COR theory posits that individuals possess an innate inclination to amass, retain, and protect resources (Hobfoll, 1989). These resources encompass tangible entities like possessions (e.g., house and car), intangible attributes such as personal characteristics (e.g., CSE, PsyCap), energies (e.g., time, energy), and conditions (e.g., organizational support, PJ fit) (Hobfoll, 2001). Thus, CSE is considered as a type of personal resource. Such key personal resource empower employees to navigate demanding scenarios, effectively manage challenges and stressors, ultimately culminating in triumphant outcomes within the professional sphere (Kim et al., 2017). Employees endowed with substantial reservoirs of these resources are adept at drawing upon them in a manner akin to having an extensive font of assets at their disposal (Hobfoll, 2011).

Building on the preceding discussion, this study posits that FLEs with elevated CSE exhibits good SRP. It is incumbent upon employees to diligently adhere to the delineated tasks within their job descriptions and earnestly attend to customer grievances. They should stand prepared to adeptly navigate challenges and address complaints. In accordance with COR theory, individuals with heightened CSE possesses an augmented reservoir of resources, affording them enhanced capacity to pursue and attain their objectives (Karatepe and Talebzadeh, 2016; Yan et al., 2022). Consequently, it is anticipated that under such circumstances, employees will manifest superior workplace performance. However, empirical studies directly testing the impact of CSE on performance-related variables are scarce. Furthermore, none of these extant studies have simultaneously assessed the collective influence of CSE components on SRP. Accordingly, we advance the following hypothesis:

*Hypothesis 1*. CSE may positively influence SRP.

### 2.2 Emotional exhaustion as a mediator (CSE → EE → SRP)

CSE constitutes a pivotal trait capturing an individual’s fundamental appraisals of their environment, thereby distinguishing individuals from one another (Anand and Mishra, 2021). Those possessing high level of CSE exhibits a positive self-regard, evincing confidence, self-assuredness, and heightened motivation (Ferris et al., 2011). Furthermore, they manifest resilience and a general contentment with both professional and personal spheres (Judge et al., 2002). Such individuals perceive themselves as proficient in problem-solving (demonstrating high self-efficacy), deserving of respect and esteem (exhibiting high self-esteem), in command of and accountable for their circumstances (embracing an internal locus of control), and inclined toward optimism while being largely impervious to doubts and anxieties (enjoying high emotional stability) (Judge and Kammeyer-Mueller, 2011). Consequently, employees characterized by positive CSE are poised to adeptly navigate and manage stressors and strains. Notably, self-esteem, generalized self-efficacy, locus of control, and emotional stability have been posited as intrinsic resources for alleviating challenges arising from such stressors and strains, particularly within the theoretical framework of COR theory. Therefore, it is conceivable that CSE may serve to mitigate EE among FLEs.

This study posits that the manifestation EE among FLEs within the workplace exerts negative effect on their SRP. The COR theory offers valuable insights into this relationship. Employees undergo stress when confronted with the perceived risk of resource depletion, actual instances of resource loss, or inadequate returns on supplementary resource investments (Hobfoll, 2001). Such stress is likely to precipitate feelings of EE. Under such exigent circumstances, FLEs may construe their resource reservoirs as insufficient to meet the demands of their work, thereby prompting the adoption of defensive strategies to avert further resource depletion. Specifically, this may encompass a reduction in organizational engagement and commitment, potentially culminating in compromised performance in job-related tasks, including SRP (Meyer et al., 2021).

While extant empirical investigations have examined the negative impact of EE on job performance, scant attention has been directed toward comprehending its deleterious influence on the SRP of FLEs. For example, Yan et al. (2023) observed a deterioration in both in-role and extra-role performance among hotel FLEs in Malaysia due to EE. Conversely, Yavas et al. (2008) reported no significant relationship between EE and job performance in their study involving hotel FLEs in Turkey. Grobelna (2021) demonstrated that EE functioned as a negative predictor of service performance among hotel FLEs in Poland. Choi C. H. et al. (2014) and Choi Y. et al. (2014) proved that EE significantly and negatively influenced SRP among FLEs in tourism industry in South Korea. In light of the foregoing discussion, we posit the following hypotheses:

*Hypothesis 2*. CSE may negatively influence EE.

*Hypothesis 3*. EE may negatively influence SRP.

*Hypothesis 4*. EE may mediate the relationship between CSE and SRP.

### 2.3 Person-job fit and service recovery performance

PJ fit is defined as the degree of consistency between employees’ personal characteristics and job characteristics. It can be further divided into the degree to which an individual’s ability fits the demands of work (demand-ability fit) and the degree to which individual’s needs fit what job supplied (need-supply fit) (Kristof-Brown et al., 2005). When employees have rich knowledge, exquisite technology, and strong personal ability that meet work requirements, it is demand-ability (D-A) fit; when work meets the expectation and demand of employees, it is need-supply (N-S) fit (Cable and DeRue, 2002). That is, PJ fit includes two dimensions respectively, D-A fit and need-supply N-S fit.

The COR theory posits that individuals desire to acquire, preserve, and maintain their limited resources. These resources are specifically classified into four primary categories: object resources, personality traits, conditional resources, and energy resources (Hobfoll, 1989). Drawing upon the conceptualization of PJ fit, it is regarded as a type of valued conditional resource (Hobfoll, 1989; Hobfoll et al., 2018). Consequently, in the present investigation, both CSE and PJ fit are construed as invaluable resources. These pivotal resources empower employees to adeptly navigate challenging circumstances, effectively cope with adversity and stress, ultimately culminating in workplace success (Kim et al., 2017). Simply speaking, such resources contribute to positive outcomes, as employees endowed with abundant resources possess an extensive reservoir of resources from which to draw (Hobfoll, 2011).

Of particular relevance is the facet of PJ fit, specifically D-A fit, which pertains to an individual’s congruence with the requirements of a specific job (Kristof-Brown, 2000). Employees possessing requisite abilities, knowledge, and skills commensurate with job prerequisites are anticipated to demonstrate enhanced job performance (Kristof-Brown, 2000). Therefore, it stands to reason that individuals with high levels of PJ fit will exhibit superior job performance compared to their counterparts with lower PJ fit (Yan et al., 2022). PJ fit is deemed indispensable for the pursuit of goals, as individuals fortified with ample resources evince confidence in successfully completing tasks and exhibit resilience in the face of obstacles and challenges (Hobfoll et al., 2018). Although the relationship between PJ fit and SRP has not been examined before, Lin et al. (2014) proved a significant and positive impact of PJ fit on job performance. Moreover, Kristof-Brown et al.’s (2005) meta-analysis revealed a more modest correlation between PJ fit and performance outcomes. Therefore:

*Hypothesis 5*. PJ fit may positively influence SRP.

### 2.4 Emotional exhaustion as a mediator (PJ Fit → EE → SRP)

PJ fit pertains to the alignment between an individual’s knowledge, skills, abilities, needs, and values with the requisites of a job position (Kristof-Brown et al., 2002). When employees perceive themselves as capable of meeting job demands, a sense of self-assurance and accomplishment ensues. Additionally, a state of diminished stress prevails when employees discern a close congruence between their inherent abilities and those necessitated by the job (Choi et al., 2017).

EE is defined as the feeling of being emotionally overextended and exhausted by one’s work (Maslach and Jackson, 1981). Service employees are entrusted with the responsibility of cultivating customer satisfaction and loyalty, a mandate that imposes a diverse range of cognitive, emotional, and behavioral demands (Cai and Chi, 2018). Notably, service employees are particularly susceptible to emotional labor, a phenomenon wherein organizations expect them to exhibit emotions that align with organizational objectives during interactions with customers (Rughoobur-Seetah, 2023). This requirement can result in job alienation, particularly when mandated emotional expressions deviate from the employee’s authentic sentiments (Amissah et al., 2022). In the service industry, the persistent challenges and heightened customer expectations can lead to both physical and emotional depletion (Choi C. H. et al., 2014). Indeed, employees in the hospitality and tourism sector frequently contend with negative customer reactions and verbal aggression, further augmenting their susceptibility to EE (Pu et al., 2022).

This study adopts COR theory to elucidate the impact of PJ fit on EE, subsequently influencing SRP. Specifically, COR theory suggests that an employee’s perception of possessing adequate conditional resources is inversely associated with strain such as EE. This in turn, augments their capacity for effective SRP (Mansour, 2023). We contend that such perceptions are more likely to manifest when an employee perceives their competencies as commensurate with job expectations — indicative of a high level of PJ fit. Hence, we propose the following hypotheses:

*Hypothesis 6*. PJ fit may negatively influence EE.

*Hypothesis 7*. EE may mediate the relationship between PJ fit and SRP.

### 2.5 Organizational dehumanization as a moderator

OD emanates from the realm of social psychology and is characterized by an employee’s perception of being objectified by their employing organization, thereby being deprived of personal subjectivity, and reduced to a mere instrument or tool for the attainment of the organization’s targets (Bell and Khoury, 2011; Gip et al., 2023; Lazaroiu and Rogalska, 2023). Koeske and Koeske’s (1993) stressor–strain–outcome (SSO) model serves as the theoretical framework for comprehending the moderating influence of OD, where OD is a job stressor, EE represents a job strain, and SRP stands as a job outcome variable (Choi C. H. et al., 2014). Aligning with the tenets of the positive stressor–strain relationship posited by SSO model, this study advances the proposition that the greater the degree of encountered OD by FLEs, the higher their levels of EE will be. Additionally, this study contends that FLEs’ experience of EE in the workplace exerts an adverse influence on their SRP, consistent with the negative strain-outcome relationship delineated within the SSO framework. Thus, this study posits that OD may play an important role in moderating the relationship between CSE, PJ fit and EE while also serving as a boundary condition affecting the indirect relationship among CSE, PJ fit and SRP. Accordingly, we hypothesize:

*Hypothesis 8*. OD may moderate the relationship between CSE and EE.

*Hypothesis 9*. The indirect effect of CSE on SRP via EE may be conditional on OD.

*Hypothesis 10*. OD may moderate the relationship between PJ fit on EE.

*Hypothesis 11*. The indirect effect of PJ fit on SRP via EE may be conditional on OD.

Hotel FLEs may indeed possess commendable personal characteristic and conditional resources, such as CSE and PJ fit. However, when they experience OD, they will tend to partake in lower levels of SRP to protect existing resources compared to those who do not experience OD. This protective inclination arises from a potential loss of motivation stemming from the depletion of existing resources, consequently impeding their capacity to effectively engage in job-related tasks, including SRP (Gip et al., 2023). Thus, this study posits that OD assumes a pivotal role in moderating the association between CSE, PJ fit and SRP. Accordingly, we hypothesize:

*Hypothesis 12*. OD may moderate the relationship between CSE and SRP.

*Hypothesis 13*. OD may moderate the relationship between PJ fit and SRP.

**Figure 1:**
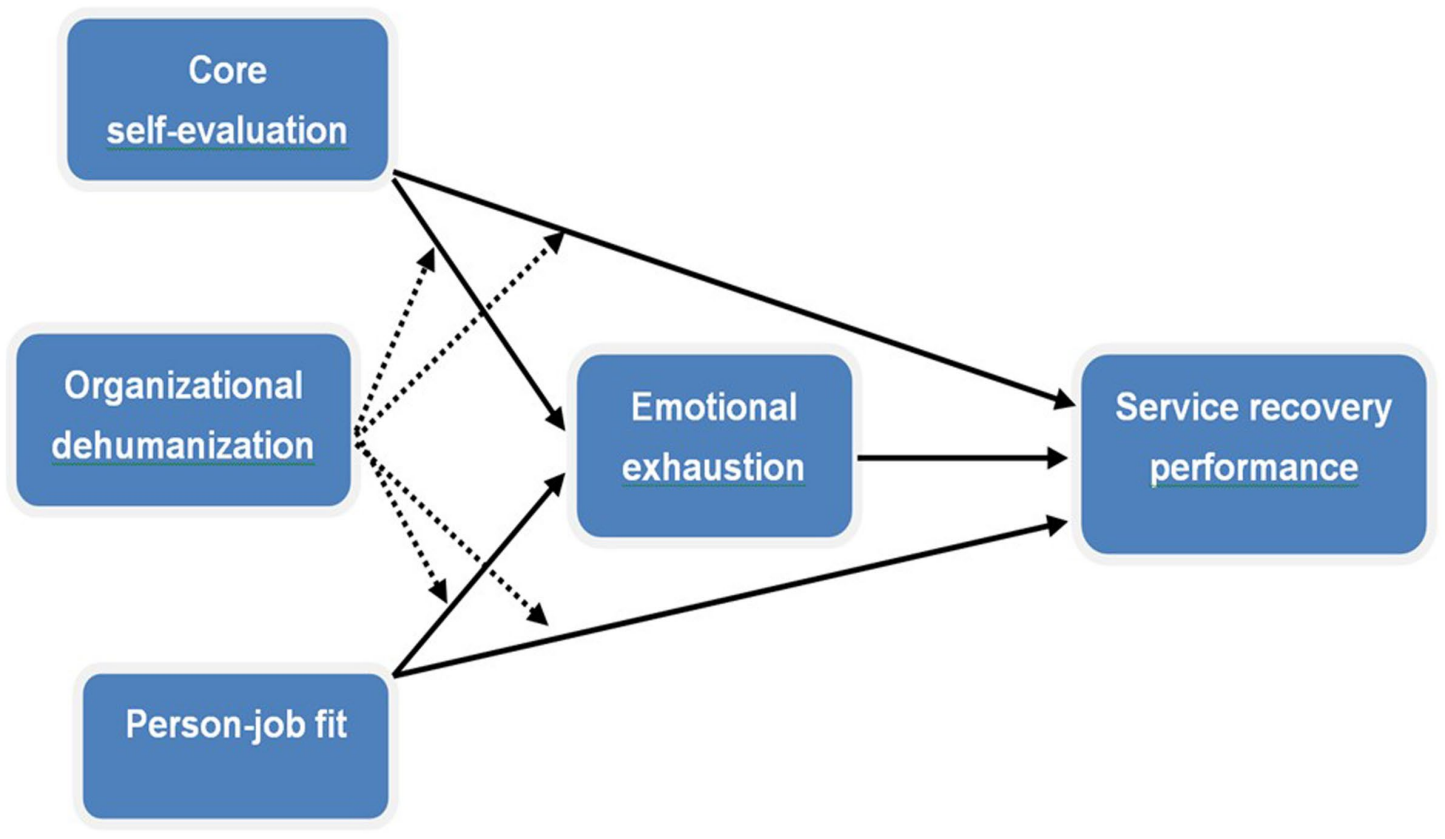
The conceptual framework.

The framework and hypotheses of the current study have been shown in Figure 1.

## 3 Methods

### 3.1 Sample and procedures

We randomly selected 12 international five-star hotels in three cities. Subsequently, nine hotels expressed consent to participate in the study. Data were meticulously gathered from four frontline departments, encompassing front office, food and beverage, housekeeping, and marketing divisions within each hotel. The research methodology involved the deliberate application of a purposive sampling technique, coupled with the administration of online survey questionnaires. The data acquisition process was structured across two distinct waves with a two-week interval. In the initial wave, we engaged with human resource directors or managers from the hotels to provide a comprehensive overview of our research objectives. Concurrently, we distributed questionnaires to 270 FLEs employed across the nine participating hotels. These questionnaires encompassed a rudimentary introduction outlining the project’s objectives, methodology and information about the assurance of confidentiality (Podsakoff et al., 2003), coupled with a survey encapsulating respondents’ demographic particulars, CSE, PJ fit, EE, and OD. Ultimately, we received a total of 238 meticulously completed surveys. Subsequently, in the second wave, which transpired two weeks later, we dispatched a follow-up questionnaire to the immediate supervisors of FLEs who had effectively completed the initial phase. They were solicited to provide assessments of their subordinates’ SRP. We garnered responses from 32 supervisors, pertaining to a cumulative total of 212 FLEs. On average, each supervisor rated five to eight their subordinates. This engendered an impressive overall response rate of 78.5%. It is noteworthy that each questionnaire was endowed with a distinctive identifier code, facilitating the seamless correlation of respondents across the two waves of data collection.

### 3.2 Measurement

In this research, we selected mature scales, which have been widely utilized in the hospitality industry to ensure the reliability and effectiveness of the measurement variables. In accordance with Brislin’s (1970) recommendation, the back-translation method was diligently employed to ensure linguistic fidelity in all measurements. Additionally, the translated instruments underwent a rigorous validation process, which involved scrutiny by two departmental managers of a hotel to fortify their psychometric integrity. Based on the following five measurement tools, totally 43 items, were included in the initial model.

#### 3.2.1 Core-self evaluation scale

The assessment of CSE was conducted employing Judge et al.’s (2005) well-established Core-Self Evaluation Scale (CSES), which encompasses 12 items gaging the four foundational facets of CSE: locus of control, generalized self-efficacy, self-esteem and neuroticism (emotional stability). A representative item for this construct is, “I am capable of coping with most of my problems.” The Cronbach’s α coefficient for this scale was 0.956.

#### 3.2.2 PJ Fit scale

Measurement of PJ fit was predicated on the two-dimensional scale formulated by Cable and DeRue (2002), encompassing N-S fit and D-A fit, each comprised of three questions. An illustrative item reads, “The attributes that I look for in a job are fulfilled very well by my present job.” The Cronbach’s α coefficient for this scale was 0.861.

#### 3.2.3 Emotional exhaustion scale

EE was measured employing a scale with eight items developed by Maslach and Jackson (1981), including an item such as “I feel frustrated with my job.” The Cronbach’s α coefficient for this scale was 0.924.

#### 3.2.4 Organizational dehumanization scale

To assess OD, 11 items were drawn from Caesens et al. (2017), featuring a sample item is “My organization makes me feel that one worker is easily as good as any other.” The Cronbach’s α coefficient for this scale was 0.948.

#### 3.2.5 Service recovery performance scale

SRP was evaluated using a six-item measure developed by Lin (2010), with a sample item “This employee is able to properly handle dissatisfied customers.” The Cronbach’s α coefficient for this scale was 0.919.

All variables were rated on a five-point Likert-type scale, ranging from strongly disagree (1) to strongly agree (5). Given previous research indicating significant impacts of FLEs’ demographic attributes on service performance, gender, age, educational background, tenure, and hotel department were included as control variables (Lyu et al., 2016; Menicucci et al., 2019).

### 3.3 Data analytic strategy

The current research utilized SPSS 25.0 for the analysis of the descriptive statistics and correlations among constructs. Confirmatory factor analysis (CFA) was conducted with AMOS 25.0 to test the measurement model and convergent and discriminant validity (Fornell and Larcker, 1981). The hypotheses were examined with PROCESS (version 3.4) (Hayes, 2017). To be specific, the mediating (Model 4), moderating (Model 1), and moderated mediation (Model 8) effects were evaluated.

## 4 Results

### 4.1 Respondent profile

The demographic characteristics of the sample are shown in Table 1. Nearly two thirds of the participants were female (63.2%). Regarding age, the largest group was those 21–30 years old (46.2%), followed by those 31–40 (33.0%), and those 41 years old and above (15.1%). In relation to educational background, 8.5% held a middle school diploma or below, 19.3% possess a high school diploma, 42.5% had an associate degree, and 29.7% achieved a bachelor’s degree or above. The largest group of respondents worked in the department of food and beverage (43.4%), followed by housekeeping (27.8%), and front office (27.0%). Most of them had 1–3 years of working experience in their hotels (39.2%).

**Table 1:** Demographic profile of the respondents (*n* = 212).

Demographic variables	Frequency	(%)
*Gender*		
Male	78	36.8%
Female	134	63.2%
*Age*		
20 years and below	12	5.7%
21–30 years	98	46.2%
31–40 years	70	33.0%
41 years and above	32	15.1%
*Education*		
Middle school and below	18	8.5%
High school	41	19.3%
Associate degree	90	42.5%
Bachelor degree and above	63	29.7%
*Tenure*		
1 year and below	57	26.9%
1–3 years	83	39.2%
4–6 years	48	22.6%
6 years above	24	11.3%
*Department*		
Food and beverage	92	43.4%
Housekeeping	59	27.8%
Front office	55	27.0%
Marketing	6	2.8%

### 4.2 Common method bias

In this research, we assured each respondent the anonymity of the survey and emphasized that there were no right or wrong answers, and we asked the respondents to answer questions as honestly as possible. Since self-report questionnaires may cause common method variance issues, the Harman single-factor test was conducted (Podsakoff et al., 2012). The result showed that the cumulative percent of first factor was 21.237% (less than the critical value of 40%), which implied that no single factor was apparent in the un-rotated factor structure. Hence, common method bias was not a serious issue in this study.

### 4.3 Measurement model

CFA was employed before testing the research hypotheses. In Table 2, CFA results exhibited a good model that fits the data (*x*^2^/df = 1.451, NFI = 0.970, RMSEA = 0.046, CFI = 0.973). Cronbach’s alpha coefficients and composite reliability (CR) are greater than the recommended thresholds for each variable. The average variance extracted (AVE) values were found to be above 0.5 for each construct, achieving convergent validity (Bagozzi and Yi, 1988). Additionally, as demonstrated in Table 3 the square roots of AVE were larger than the bivariate correlation coefficients, providing robust evidence for discriminant validity (Henseler et al., 2015).

**Table 2:** Results of reliability and convergent validity of full measurement model.

Variables and items	Standardized loadings	CR	AVE	Cronbach α
*Core self-evaluations*		0.957	0.847	0.956
SEv	0.920			
GSEv	0.918			
LOCv	0.904			
NEUv	0.938			
*Person-job fit*		0.862	0.512	0.861
PJ1	0.754			
PJ2	0.735			
PJ3	0.736			
PJ4	0.586			
PJ5	0.720			
PJ6	0.749			
*Emotional exhaustion*		0.925	0.607	0.924
EE1	0.794			
EE2	0.853			
EE3	0.703			
EE4	0.752			
EE5	0.788			
EE6	0.691			
EE7	0.755			
EE8	0.878			
*Service recovery performance*		0.92	0.657	0.919
SRP1	0.776			
SRP2	0.815			
SRP3	0.845			
SRP4	0.766			
SRP5	0.851			
SRP6	0.808			
*Organizational dehumanization*		0.925	0.536	0.948
OD1	0.998			
OD2	0.662			
OD3	0.615			
OD4	0.661			
OD5	0.683			
OD6	0.655			
OD7	0.653			
OD8	0.994			
OD9	0.679			
OD10	0.621			
OD11	0.699			

CR, Composite reliability; AVE, Average variance extracted.

**Table 3:** Mean, standard deviation, inter-correlations of variables and discriminant validity.

Variables	Mean	SD	1	2	3	4	5	6
1. CSE	3.54	0.969	***0.92***					
2. PJ fit	3.50	0.715	0.45***	***0.72***				
3. EE	3.53	0.975	−0.32 ***	−0.40***	***0.78***			
4. SRP	3.68	0.977	0.54***	0.39***	−0.58***	***0.81***		
5. OD	3.65	0.861	−0.25***	−0.20*	0.45***	−0.45***	***0.73***	

*N*, 212; SD, standard deviation; CSE, core self-evaluations; PJ fit, person-job fit; EE, emotional exhaustion; SRP, service recovery performance; OD, organizational dehumanization.

*p < 0.05; ***p < 0.001; the square root of AVE for each variable is shown with bold italics.

### 4.4 Tests of mediation

As presented in Table 4, CSE (β = 0.40, *p* < 0.001) positively influenced SRP, thereby supporting Hypotheses 1. CSE (β = −0.30, *p* < 0.001) was negatively associated with EE, supporting Hypotheses 2. Moreover, EE (β = −0.45, *p* < 0.001) has a negative relationship with SRP. Thus, Hypothesis 3 was confirmed. Furthermore, the indirect effect of CSE on SRP through EE was significant, with 95% CI = (0.07, 0.20) which does not include 0. Hence, H4 was accepted.

**Table 4:** Mediation results (CSE → EE → SRP) (PROCESS: model 4).

Model	Coefficient	SE	*t*-value	*p*-value	LLCI	ULCI
*Model 1:* *mediator variable model* *Outcome: EE*						
CSE	−0.30	0.07	−4.54	<0.001	−0.43	−0.17
Gender	−0.06	0.13	−0.43	>0.05	−0.32	0.20
Age	−0.20	0.11	−1.83	>0.05	−0.41	0.02
Educational background	−0.01	0.09	−0.14	>0.05	−0.19	0.16
Tenure	0.01	0.09	0.16	>0.05	−0.16	0.19
Department	−0.07	0.07	−0.93	>0.05	−0.21	0.07
*Model 2:* *outcome variable model* *Outcome: SRP*						
CSE	0.40	0.06	6.53	<0.001	0.28	0.52
EE	−0.45	0.06	−7.13	<0.001	−0.57	−0.32
Gender	0.12	0.12	0.98	>0.05	−0.12	0.35
Age	0.12	0.10	1.27	>0.05	−0.07	0.31
Educational background	−0.06	0.08	−0.70	>0.05	−0.21	0.10
Tenure	−0.04	0.08	−0.44	>0.05	−0.19	0.12
Department	0.04	0.06	0.62	>0.05	−0.09	0.17

*Bootstrapping results for the indirect effect*				
	Effect	BootSE	LL 95% CL	UL 95% CI
Indirect effect of CSE on SRP via EE	0.13	0.03	0.07	0.20

*N*, 212; CSE, core self-evaluations; EE, emotional exhaustion; SRP, service recovery performance, LL, Lower limit; UL, Upper limit; CI, Confidence interval.

As can be seen in Table 5, PJ fit (β = 0.29, *p* < 0.001) had positive impact on SRP, thereby supporting Hypotheses 5. PJ fit (β = −0.48, *p* < 0.001) was negatively associated with EE, supporting Hypotheses 6. Moreover, the indirect effect of PJ fit on SRP through EE was significant, with 95% CI = (0.14, 0.36) which does not include 0. Thus, H7 was accepted.

**Table 5:** Mediation results (PJ fit → EE → SRP) (PROCESS: model 4).

Model	Coefficient	SE	*t*-value	*p*-value	LLCI	ULCI
*Model 1:* *mediator variable model* *Outcome: EE*						
PJ fit	−0.48	0.10	−5.10	<0.001	−0.67	−0.30
Gender	−0.09	0.13	−0.70	>0.05	−0.35	0.17
Age	−0.15	0.11	−1.42	>0.05	−0.36	0.06
Educational background	−0.02	0.09	−0.19	>0.05	−0.19	0.16
Tenure	0.02	0.09	0.02	>0.05	−0.17	0.17
Department	−0.09	0.07	−1.31	>0.05	−0.23	0.05
*Model 2:* *Outcome variable model* *Outcome: SRP*						
PJ fit	0.29	0.10	2.98	<0.01	0.10	0.49
EE	−0.50	0.07	−7.36	<0.001	−0.64	−0.37
Gender	0.18	0.12	1.42	>0.05	−0.07	0.42
Age	0.08	0.10	0.82	>0.05	−0.12	0.28
Educational background	−0.03	0.08	−0.35	>0.05	−0.19	0.13
Tenure	−0.05	0.08	−0.60	>0.05	−0.21	0.11
Department	0.04	0.07	0.64	>0.05	−0.09	0.18

*Bootstrapping results for the indirect effect*				
	Effect	BootSE	LL95%CL	UL95%CI
Indirect effect of PJ fit on SRP via EE	0.24	0.06	0.14	0.36

*N*, 212; PJ fit, person-job fit; EE, emotional exhaustion; SRP, service recovery performance; LL, Lower limit; UL, Upper limit; CI, Confidence interval.

### 4.5 Tests of moderated mediation

**Figure 2:**
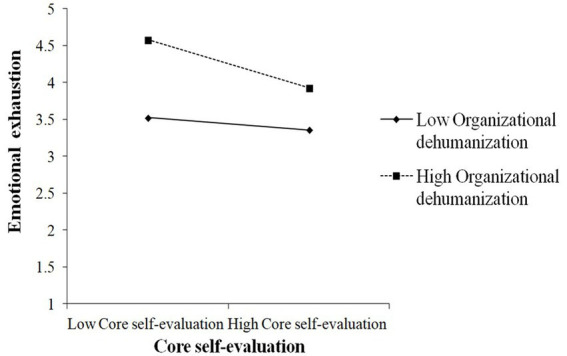
The interactive effects of core self-evaluations and organizational dehumanization on emotional exhaustion.

As demonstrated in Table 6, OD significantly moderated the impact of CSE on EE (β = −0.14, *p* < 0.05). To better understand this moderating effect, we created a visual representation by calculating the slopes at 1 standard deviation above and below the mean (Aiken and West, 1991). Figure 2 shows the interaction pattern. Thus, OD significantly moderated the relationship between CSE and EE and hypothesis 8 was supported. The findings presented in Table 6 indicated that the conditional indirect effect was weaker in the low OD condition (β = 0.03 CI −0.01, 0.09) and stronger in the high condition (β = 0.10, CI 0.04, 0.17). Additionally, the index of moderated mediation was found to be statistically significant (index = 0.04, CI 0.01, 0.08). Hence, Hypothesis 9 was supported.

**Table 6:** Results of the moderated mediation analysis (PROCESS: model 8).

Model	Coefficient	SE	*t*-value	*p*-value	LLCI	ULCI
*Model 1:* *mediator variable model* *Outcome: EE*						
CSE	−0.21	0.06	−3.50	<0.01	−0.48	−0.24
OD	0.46	0.07	6.80	<0.001	0.33	0.59
CSE × OD	−0.14	0.06	−2.53	<0.05	−0.25	−0.03
Gender	0.03	0.12	0.28	>0.05	−0.20	0.27
Age	−0.15	0.10	−1.55	>0.05	−0.34	0.04
Educational background	0.00	0.08	0.00	>0.05	−0.16	0.16
Tenure	0.02	0.08	0.21	>0.05	−0.14	0.17
Department	−0.02	0.06	−0.35	>0.05	−0.15	0.10
*R*^2^ = 0.30						
*Model 2:* *Outcome variable model* *Outcome: SRP*						
CSE	0.40	0.06	6.74	<0.001	0.28	0.51
EE	−0.29	0.07	−7.13	<0.001	−0.42	−0.16
OD	−0.34	0.07	−4.92	<0.001	−0.48	−0.21
CSE *×* OD	0.17	0.05	3.16	<0.01	0.06	0.27
Gender	0.06	0.11	0.53	>0.05	−0.16	0.28
Age	0.12	0.09	1.31	>0.05	−0.06	0.30
Educational background	−0.07	0.07	−0.90	>0.05	−0.21	0.08
Tenure	−0.03	0.07	−0.47	>0.05	−0.18	0.11
Department	0.02	0.06	0.28	>0.05	−0.10	0.14
*R*^2^ = 0.50						

*Bootstrapping results for conditional indirect effect*						
OD (low)	0.03	0.03			−0.01	0.09
OD (High)	0.10	0.04			0.04	0.17
Index of moderated mediation	0.04	0.02			0.01	0.08

*N*, 212; CSE, core self-evaluations; EE, emotional exhaustion; SRP, service recovery performance; OD, organizational dehumanization; LL, Lower limit; UL, Upper limit; CI, Confidence interval.

**Figure 3:**
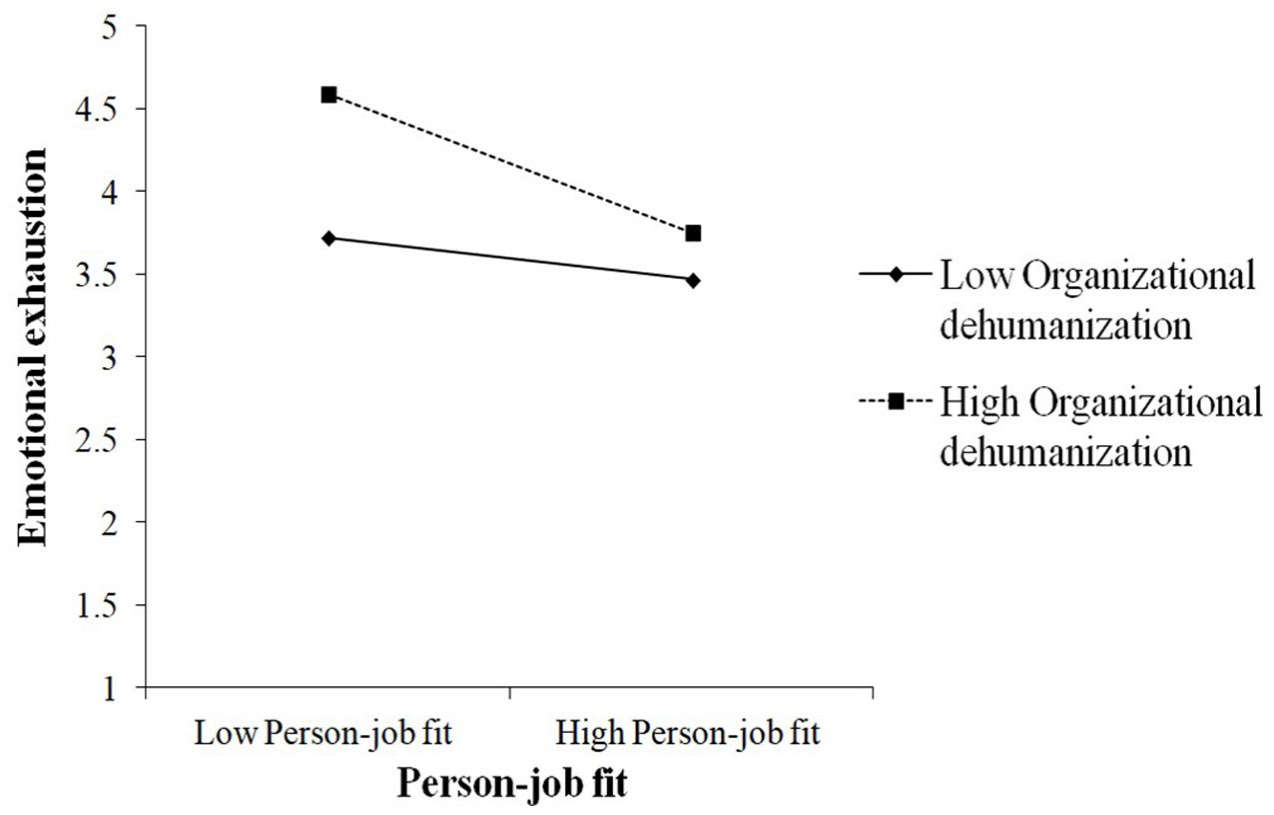
The interactive effects of person-job fit and organizational dehumanization on emotional exhaustion.

As shown in Table 7, OD significantly moderated the association between PJ fit and EE (β = −0.25, *p* < 0.01). To better understand this moderating effect, we created a visual representation by calculating the slopes at 1 standard deviation above and below the mean (Aiken and West, 1991). Figure 3 shows the interaction pattern. Thus, OD significantly moderated the relationship between PJ fit and EE and hypothesis 10 was supported. The findings presented in Table 7 indicated that the conditional indirect effect was weaker in the low OD condition (β = 0.03, CI −0.09, 0.16) and stronger in the high condition (β = 0.18, CI 0.08, 0.31). In addition, the index of moderated mediation was found to be statistically significant (index = 0.08, CI 0.01, 0.19). Thus, Hypothesis 11 was confirmed.

**Table 7:** Results of the moderated mediation analysis (PROCESS: model 8).

Model	Coefficient	SE	*t*-value	*p*-value	LLCI	ULCI
*Model 1:* *mediator variable model* *Outcome: EE*						
PJ fit	−0.31	0.09	−3.51	<0.01	−0.49	−0.14
OD	0.43	0.07	6.58	<0.001	0.30	0.56
PJ fit *×* OD	−0.25	0.09	−2.80	<0.01	−0.42	−0.07
Gender	0.02	0.12	0.18	>0.05	−0.21	0.25
Age	−0.11	0.10	−1.18	>0.05	−0.30	0.08
Educational background	0.01	0.08	−0.11	>0.05	−0.16	0.14
Tenure	0.00	0.08	0.00	>0.05	−0.15	0.15
Department	−0.05	0.06	−0.80	>0.05	−0.18	0.08
*R*^2^ = 0.33						
*Model 2:* *Outcome variable model* *Outcome: SRP*						
PJ fit	0.25	0.10	2.59	<0.05	0.06	0.43
EE	−0.34	0.07	−4.63	<0.001	−0.48	−0.19
OD	−0.36	0.08	−4.80	<0.001	−0.51	−0.21
PJ fit × OD	0.13	0.09	2.37	<0.05	0.06	0.31
Gender	0.12	0.12	0.96	>0.05	−0.12	0.36
Age	0.08	0.10	0.85	>0.05	−0.11	0.28
Educational background	−0.04	0.08	−0.52	>0.05	−0.20	0.12
Tenure	−0.04	0.08	−0.53	>0.05	−0.20	0.12
Department	0.02	0.07	0.25	>0.05	−0.11	0.15
*R*^2^ = 0.40						

*Bootstrapping results for conditional indirect effect*						
OD (low)	0.03	0.06			−0.09	0.16
OD (High)	0.18	0.06			0.08	0.31
Index of moderated mediation	0.08	0.05			0.01	0.19

*N*, 212; PJ fit, person-job fit; EE, emotional exhaustion; SRP, service recovery performance; OD, organizational dehumanization; LL, Lower limit; UL, Upper limit; CI, Confidence interval.

**Figure 4:**
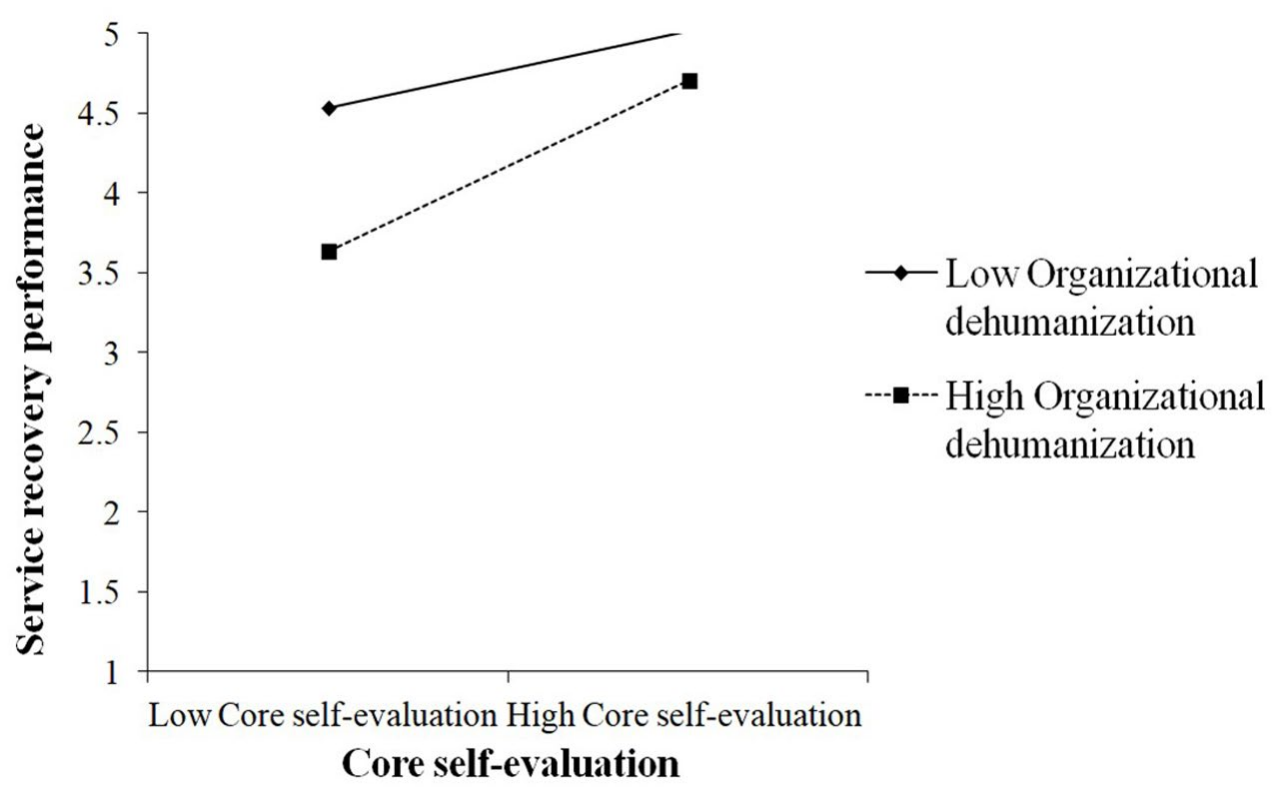
The interactive effects of core self-evaluations and organizational dehumanization on service recovery performance.

**Figure 5:**
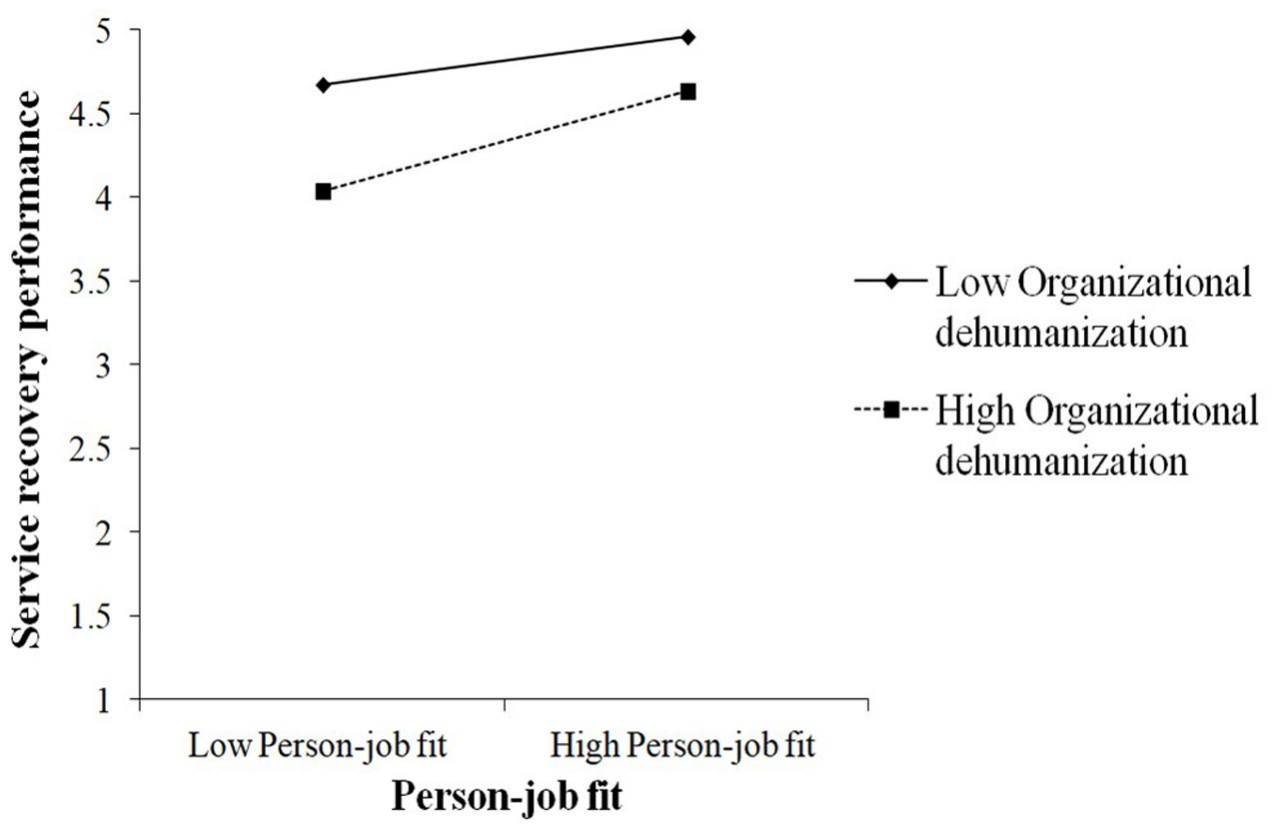
The interactive effects of person-job fit and organizational dehumanization on service recovery performance.

Furthermore, OD significantly moderated the direct association between CSE and SRP (β = 0.17, *p* < 0.01). This interaction pattern is illustrated in Figure 4. Thus, hypothesis 12 was supported. Besides, OD significantly moderated the direct association between PJ fit and SRP (β = 0.25, *p* < 0.05). Figure 5 displays the interaction pattern, affirming hypothesis 13.

## 5 Discussion

In this study, we utilized a moderated mediation model shown in Figure 1 to present the relationships between CSE, PJ fit and SRP by incorporating EE and OD. Based on COR theory, this model is the first to examine the direct effect of CSE and PJ fit on SRP in the context of hospitality, as well as the indirect effect of CSE and PJ fit on the abovementioned outcome through EE, with OD as the moderator. However, most of prior studies only focused on simple effect of personality traits (such as CSE, PJ fit) on SRP (Yavas et al., 2010; Oentoro et al., 2016), without in-depth exploration on underlying and conditional mechanisms behind it. Therefore, there is a necessity to conduct this study.

### 5.1 Theoretical implications

The present study makes a significant contribution to the hospitality literature by investigating the influence of CSE and PJ fit on FLEs’ SRP. To the best of our knowledge, there is no prior research that has directly examined these relationships, particularly in the context of hospitality industry. Furthermore, our study stands out for incorporating personal and conditional resources into the model and it reveals that these resources exert differing effects on the outcome, which is also a noteworthy finding.

Second, given that FLEs’ SRP holds paramount importance in the fiercely competitive hospitality sector, numerous scholars have increasingly focused on its antecedents (Yao et al., 2019). However, the extant literature has predominantly centered on job-specific demands and resources, often ignoring the fact that FLEs’ personal resources and conditional resources may also significantly influence SRP. In light of this, our study addressed a critical gap by introducing CSE and PJ fit into the current understanding of how personal and conditional resources have impact on SRP.

Third, we sought to uncover a crucial underlying mechanism of SRP by including EE as a mediating variable, providing detailed insights into the process through which CSE and PJ fit influence FLEs’ SRP. The COR theory and stressor–strain–outcome model offer a valuable guideline for comprehending such a relationship. Both CSE and PJ fit relieve EE, as FLEs’ perceptions of having sufficient resources lead to reduced stress and EE. EE in the workplace can have a deleterious impact on their SRP. Employees may undergo stress when they perceive a risk of losing resources, face actual resource loss, or receive insufficient returns on their investments of resources (Hobfoll, 2001). This stress is likely to culminate in EE. Specifically, after a substantial investment of effort in their work, FLEs may come to believe that their resources are no longer adequate to meet the demands of their work. As a result, when employees’ emotional resources for handling job demands are depleted, they may face challenges in carrying out job-related tasks successfully such as SRP (Meyer et al., 2021).

Fourth, through examining the moderating role of OD, this study elucidated the boundary condition in the process wherein CSE and PJ fit influence EE and SRP. Muhammad and Sarwar (2021) argued that the concept of OD was still in its early stage in organizational behavior. Limited literature has considered that OD as an antecedent has negative impact on attitudinal and behavioral outcomes such as job satisfaction, affective commitment, deviation, psychosomatic strains and turnover intention (Lagios et al., 2021; Muhammad and Sarwar, 2021; Nguyen and Stinglhamber, 2021). This study is also the first to incorporate OD as a moderating variable to illustrate the work-stress process based on COR theory.

### 5.2 Practical implications

In addition to the theoretical significance, this study holds crucial implications for practitioners. First, hotel management should acknowledge the vital role of personal and conditional resources and incorporate assessments of candidates’ CSE and PJ fit in the process of selection. With reliable measurement tools like CSE and PJ fit questionnaires (Cable and DeRue, 2002; Judge et al., 2005) readily available, organizations can identify candidates with ample personal and conditional resources. This implication empowers managers to recruit individuals possessing positive personal attributes for the appropriate positions.

Second, hospitality FLEs need frequently adjust their emotions and express the emotions to align with organizational demands. FLEs frequently interact emotionally with customers, potentially leading to EE. High levels of EE among employees have negative effects on organizational outcomes, as it may result in diminished positive attitudes and reduced performance (Rathi and Lee, 2016). The current study has proved that if FLEs’ resources are depleted in coping with their job demands, their ability to execute job-related tasks, such as SRP, may be compromised (Meyer et al., 2021). Hence, the HR department should recognize that personal and conditional resources are adaptable and can be cultivated within an individual (Luthans et al., 2007). Various face-to-face and web-based interventions are available to improve FLEs’ CSE and PJ fit. Consequently, hotel management can proactively implement training programs to sustain high levels of CSE and PJ fit among FLEs. Through these programs, FLEs can learn effective strategies to protect, preserve and accumulate their personal and conditional resources.

Third, OD has low intensity compared with overt physical aggression, and is often overlooked by practitioners. The findings on OD as a stressor demonstrate its detrimental effect on the preservation of personal and conditional resources, particularly in relation to EE. Therefore, managers should devise interventions that empower hotel FLEs, making them aware that they are valued as individuals and not merely as replaceable instruments. It is imperative for hotels to recognize that prioritizing the well-being of their employees as human beings precedes concerns about their performance. Managers can foster a sense of belonging and support by implementing HR practices like reducing workload, enhancing their perceptions of job security and offering ample training and development chances for their personal growth and advancement. To counteract feelings of dehumanization among FLEs, conferences, workshops, teambuilding activities involving active interaction across various levels of management can be highly effective. These initiatives can promote engagement, respect and civility in the workplace, ultimately reducing perceived stressors among FLEs (Muhammad and Sarwar, 2021).

Finally, management should be responsible for ensuring FLEs play a central role in the process of service delivery. If management identifies FLEs who struggle with addressing customer issues and are hesitant to enhance their SRP, it may be advisable to consider replacing them with individuals who exhibit high levels of CSE and PJ fit. In conclusion, the practices outlined above are likely to empower management in acquiring FLEs high on CSE and PJ fit, mitigating their EE and thereby improving SRP.

## 6 Limitations and future research

While the present study provides valuable insights for both academia and practice, it is imperative to acknowledge certain limitations. First, the cross-sectional design employed in the current research constrains its capacity to definitely establish causal relationships. Despite we have made efforts to prevent or reduce the likelihood of CMB through multiple procedural approaches, its potential influence on the results cannot be entirely ruled out (Podsakoff et al., 2003). For instance, CSE, PJ fit, EI and OD were all assessed by the same respondents during the same time wave. In forthcoming investigations, alternative methodologies such as longitudinal or experimental studies are recommended to avoid or minimize CMV (Podsakoff et al., 2003). Second, we only focused on two types of resources, namely CSE and PJ fit, in relation to SRP in the hospitality industry. It is conceivable that other types of personal or conditional resources such as psychological capital, emotional intelligence, and PO fit, may also exert similar influence on FLEs’ SRP. Hence, future research should explore and compare the impacts of other types of resources on the outcome. Thirdly, the current study exclusively sampled FLEs in China, potentially limiting the generalizability of the findings. It is recommended that future research replicate this one within Western contexts to ascertain the robustness and consistency of the results. Finally, given the data may be nested within supervisors because FLEs shared supervisors, a multilevel empirical study within this topic can be conducted in future studies.

## 7 Conclusion

This moderated mediation model represents a notable advancement, building on previous research pertaining to CSE, PJ fit and SRP by incorporating EE and OD. This framework stands out as the first to empirically validate both the direct impact of CSE and PJ fit on SRP, and the indirect influence of CSE and PJ fit on the outcome through EE, with OD serving as the moderator. The results unequivocally demonstrated that CSE and PJ fit led to an increase in SRP and such relationships were partially mediated by EE and moderated by OD (Preacher and Hayes, 2008). Moreover, our findings shed light on the fact that OD not only intensified the association between CSE, PJ fit and EE but also bolstered the mediating effect of EE between CSE, PJ fit, and the aforementioned outcome.

## Data availability statement

The original contributions presented in the study are included in the article/supplementary material, further inquiries can be directed to the corresponding author.

## Ethics statement

The studies involving humans were approved by Human Subjects Ethics Committee of Qingdao Vocational and Technical College of Hotel Management. The studies were conducted in accordance with the local legislation and institutional requirements. The participants provided their written informed consent to participate in this study.

## Author contributions

ZZ: Conceptualization, Funding acquisition, Supervision, Writing – review & editing. ZY: Data curation, Formal analysis, Investigation, Methodology, Writing – original draft, Writing – review & editing.
